# Binding immunoglobulin protein resolves rheumatoid synovitis: a xenogeneic study using rheumatoid arthritis synovial membrane transplants in SCID mice

**DOI:** 10.1186/ar3463

**Published:** 2011-09-14

**Authors:** Kaoru Yoshida, Akira Ochiai, Hiroaki Matsuno, Gabriel S Panayi, Valerie M Corrigall

**Affiliations:** 1 Biomedical Engineering Center, Toin University of Yokohama, 1614 Kurogane-cho, Aoba-ku, Yokohama 225-8502, Japan; 2 Department of Academic Rheumatology, King's College London School of Medicine at Guy's King's and St Thomas' Hospitals, CMCBI, 1st Floor New Hunts House, Guy's Hospital Campus, London SE1 9RT, UK

## Abstract

**Introduction**

Binding immunoglobulin protein (BiP) has previously shown powerful anti-inflammatory properties in the collagen-induced arthritis (CIA) model, where a single dose of BiP has proved to be both a long-term prophylactic and therapeutic. In both CIA and human *in vitro *studies, BiP induced regulatory T cells. The present investigation looked at the anti-inflammatory effect of BiP on inflamed human synovial tissue transplanted into severe combined immunodeficient mice (SCID), a chimaeric *in vivo *model previously used to test the efficacy of biologic therapies.

**Methods**

Rheumatoid arthritis synovial membrane (RASM) was engrafted into SCID mice. Following successful engraftment, mice were intravenously injected with BiP or human serum albumin in the presence or absence of anti-IL-10 mAb. Twelve days later the grafts were removed for analysis and human cytokines in the sera were quantified by ELISA. The extent of residual inflammatory cellular infiltrate in the synovial explants was determined by weight of the explants.

**Results**

The RASM transplants from mice treated with BiP showed visual reduction in cellular infiltrate and downregulation of all quantifiable features of inflammation as assessed by the Koizumi or Rooney histological criteria. Also downregulated were HLA-DR, CD86, IL-6 and TNFα expression as assessed by immunohistology. ELISA detected significantly less human IL-6 circulating in the BiP-treated mouse serum. After removal of transplanted tissue 12 days post administration of BiP, the RASM explants from the BiP-treated SCID mice weighed significantly less, indicating a suppression of tissue inflammation. Mice given concomitant neutralising anti-IL-10 antibody and BiP showed no such suppression.

**Conclusions**

BiP has anti-inflammatory properties partially dependent on the downregulation of HLA-DR and co-stimulatory molecules and the predominant production of IL-10.

## Introduction

Rheumatoid arthritis (RA) is a chronic inflammation disease characterised by hypertrophy of the synovial membrane, ultimately causing joint damage due, in part, to the sustained production of inflammatory cytokines such as TNFα, IL-1β and IL-6. We have previously shown that binding immunoglobulin protein (BiP) downregulates both immune and inflammatory responses *in vitro *in our work with human peripheral blood mononuclear cells [1], where it attenuates TNFα production and upregulates production of IL-10, IL-1 receptor antagonist and soluble TNF receptor II. I*n vivo*, using the murine collagen-induced arthritis (CIA) model in either DBA/1 or HLA-DR1^+/+ ^transgenic mice [2], BiP has long-lasting prophylactic and therapeutic action. Importantly, the immunoregulatory function of BiP is not dependent on the continued presence of the protein, since adoptive transfer of spleen and lymph node cells from BiP-treated animals into mice with CIA could prevent or treat arthritis without further administration of BiP [2].

Biologic therapies have been successfully used in the therapy of RA over the past decade but a significant number of patients fail to respond to their treatment [3]. BiP, however, has a different mechanism of action from the currently available antibody therapies, as evident from the long-term disease remission seen in animal studies [2]. As a possible therapeutic agent in RA, however, BiP requires further validation of its anti-arthritic properties. *In vitro *human studies have established that the downregulation of HLA-DR and the co-stimulatory molecule CD86 are sensitive biomarkers of BiP activity, as is the rapid attenuation of TNFα production and increase in IL-10 production [1]. Prior to clinical studies it is necessary to confirm that these remain useful markers in a relevant model. We therefore chose a xenogeneic *in vivo *model involving transplant of human rheumatoid arthritis synovial membrane (RASM) into severe combined immunodeficient (SCID) mice. This model has been validated previously as a robust screen for therapeutic efficacy since anti-TNFα [4] and anti-soluble IL-6 receptor [5] antibodies suppress inflammation in similar models.

## Materials and methods

### Preparation of recombinant human binding immunoglobulin protein

BiP was prepared as previously described [6]. The protein purity, as assessed by polyacrylamide gel electrophoresis and silver staining, was greater than 95%. Professional assessment of endotoxin contamination showed < 0.3 endotoxin units/μg protein (Associates of Cape Cod, Liverpool, UK).

### Preparation of RASM/SCID mice

The RASM/SCID (CB.17/Icr; Charles River Japan, Tokyo, Japan) murine model was set up as described [4]. All RA patients providing tissue during knee joint replacement surgery gave fully informed written consent and the study was approved by the Research Ethics Committee of Toin University of Yokohama Project approval number I-1.

Therapeutic manipulation of the mice was undertaken only if successful engraftment had been achieved 4 weeks after transplantation. BiP (10 μg/mouse, *n *= 15) or human serum albumin (HSA) (10 μg/mouse, *n *= 15), as the control protein, were administrated intravenously either alone or in the presence or absence of anti-IL-10 antibody or isotype control antibody as required. The mice were sacrificed 12 days later and implanted tissue was removed for analysis by immunohistology and weight.

### Scoring the degree of synovial inflammation and inflammatory cell infiltrate

The degree of histological synovial inflammation of the implanted tissue was assessed as described by Koizumi and colleagues [7] or by Rooney and colleagues [8]. The scoring features included measurements of synovial hyperplasia, fibrosis, blood vessels, perivascular lymphocytes, lymphoid follicles, and diffuse infiltrating lymphocytes or synovial cells, palisading, giant cells, lymphocytes, granular tissue and fibrosis.

### Immunohistological examination

Paraffin-embedded tissue sections were used for immunostaining for CD86 and HLA-DR. Frozen tissue sections were stained for the detection of cytokines (TNFα, IL-6 and IL-10). The tissue sections were blocked for endogenous peroxidase activity with 0.3% hydrogen peroxide in methanol. Nonspecific antibody binding was blocked with 10% normal goat serum. The sections were incubated with specific antibody or normal IgG for 1 hour at 37°C. The sections incubated with anti-CD86 or HLA-DR antibody were treated with biotinylated secondary antibody, and then visualised using the Vectastatin ABC kit (Vector Laboratories, Funakoshi Co, Tokyo, Japan). A visual scoring scale of 0 to 3 was used to assess the expression of the molecules. The other sections were treated with peroxidase-conjugated anti-mouse IgG (Histofine Simple Stain MAX PO; Nichirei, Tokyo, Japan) developed with 3-amino-*n*-ethylcarbazole (Nichirei) for visualisation. Sections were counterstained with Mayer haematoxylin.

### Measurement of human IL-4, IL-6 and IL-10 in mouse serum

The serum levels of human IL-4, IL-6 and IL-10 were measured by a quantitative sandwich enzyme immunoassay technique (Quantikine HS; R&D Systems, Funakoshi Co Tokyo, Japan) according to the manufacturer's instructions.

### Statistical analysis

Data were compared using the Student *t *test and expressed as the mean ± standard deviation.

## Results and discussion

It is difficult to extrapolate from data derived either from animal models or *in vitro *human experiments to envisage how a potential therapy will act in humans. Previously, however, the RASM/SCID model has been used to predict the success of biologic therapies currently being used in the clinic for RA, such as anti-TNFα [4].

### BiP abrogates inflammation in human RASM transplanted into SCID mice

**Figure 1:**
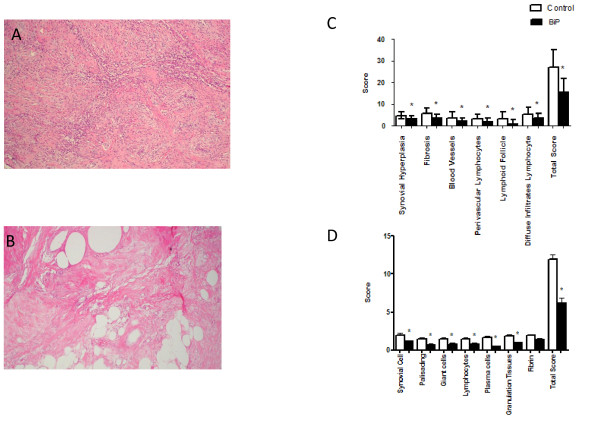
**Binding immunoglobulin protein reduces gross pathology of synovial membrane transplants**. Pieces of human synovial tissue from patients with rheumatoid arthritis were transplanted into severe combined immunodeficient mice. After successful engraftment, binding immunoglobulin protein (BiP) (10 μg/animal) or human serum albumin (HSA) (10 μg/animal) were administered intravenously, and 12 days later the tissue was removed for immunohistological examination. Representative figures of haematoxylin-stained tissue removed from **(a) **HSA-treated mice or **(b) **BiP-treated mice. Transplant samples were scored by **(c) **Rooney and colleagues' and **(d) **Koizumi and colleagues' histological criteria. Results are the mean and standard deviation of 15 animals in each group. *P < 0.02 in a comparison of BiP-treated and HSA-treated samples.

Twelve days following intravenous injection of BiP into the RASM/SCID chimaeric mice, the histological features of the RASM taken from the control mice were unchanged (Figure 1a) compared with the markedly reduced cellular infiltrate in the RASM grafts from the BiP-treated mice (Figure 1b). Histopathogical measurements of the RASM grafts from mice given intravenous BiP showed that although there was not a complete absence of inflammation it was significantly reduced compared with grafts taken from control mice (overall scores: Rooney, BiP 16 ± 6 vs. HSA 27 ± 8.2, *P *= 0.006; Koizumi, BiP 6.1 ± 2.6 vs. HSA 12 ± 2.2, *P *< 0.001; BiP *n *= 15 and HSA *n *= 14) (Figure 1c and 1d, respectively). The data from this experimental model confirm that BiP acts in an anti-inflammatory fashion and they predict a reduction in pathology *in vivo*. Indeed, the gross pathology following BiP treatment showed results similar to those seen in the same model following administration of anti-TNFα or anti-IL-6 receptor mAbs [4,5] and also the disease-modifying drug methotrexate [9].

### BiP reduces antigen presentation and inflammatory cytokines

**Figure 2:**
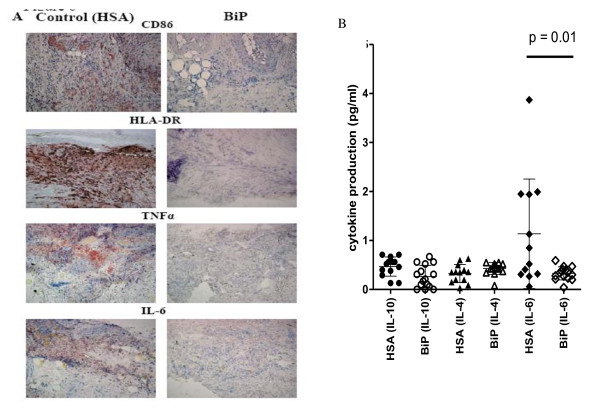
**Binding immunoglobulin protein treatment alters cell surface molecule expression and cytokine production**. Binding immunoglobulin protein (BiP) treatment alters HLA-DR and co-stimulatory molecule expression and cytokine production in the xenogeneic model of synovial membrane from rheumatoid arthritis patients transplanted into severe combined immunodeficient (SCID) mice. Pieces of human synovial tissue from patients with rheumatoid arthritis were transplanted into SCID mice. After successful engraftment, BiP (10 μg/animal) or human serum albumin (HSA) (10 μg/animal) were administered intravenously, and 12 days later tissue and serum were removed for analysis. **(a) **Representative immunohistology photomicrographs showing explants of synovial membrane taken from mice injected with HSA (left) or from mice injected with BiP (right). **(b) **Scattergram showing the concentration of human cytokines IL-4, IL-6 or IL-10 detected in the sera of mice given either HSA or BiP, as detected by ELISA. Each group contained 15 animals.

Immunohistological assessment of the RASM tissue sections showed significantly reduced expression of the co-stimulatory molecule CD86 (mean visual score ± standard error: HSA 1.86 ± 0.46, *n *= 7 vs. BiP 0.89 ± 0.35, *n *= 9; *P *= 0.05) and a trend to the downregulation of HLA-DR (HSA 1.67 ± 0.37, *n *= 9 vs. BiP 1.37 ± 0.37, *n *= 8, *P *< 0.06 (NS)) (Figure 2a), thus reducing the capacity for antigen presentation by monocytes/macrophages/dendritic cells. This observation complements our *in vitro *data showing that BiP has a significant inhibitory effect on HLA-DR expression and downregulates CD86 expression by human peripheral blood monocytes [1] and monocyte-derived dendritic cells [10]. One mechanism of action by which BiP might reduce T-cell activation, and thus inflammation, is therefore by restricting the antigen-presenting abilities of monocytes [1].

Additionally, a profound suppression of inflammatory cytokines TNFα and IL-6 was noted in RASM retrieved from the BiP-treated animals (Figure 2a). The suppression of TNFα in the RASM by BiP is particularly pertinent in diseased tissue where TNFα is known to be the major inflammatory mediator, and gives credence to such disease-related changes noted in *in vitro *human peripheral blood mononuclear cell cultures.

Only human IL-6 was produced at levels detectable in the sera of control mice injected with HSA, but not in the sera of mice given intravenous BiP. There was a significant reduction in background levels of circulating human IL-6 in these mice (HSA 1.13 ± 1.1 pg/ml vs. BiP 0.32 ± 0.13 pg/ml, *P *= 0.01) (Figure 2b). This is an important correlation. Since IL-6 is a pleiotropic cytokine this inhibition may have important consequences if translated to RA patients receiving BiP, including: reduced stimulus for the acute-phase response, leading to a reduction in C-reactive protein and the erythrocyte sedimentation rate; inhibition of development of Th17 cells, which are important proinflammatory cells in RA [11] for which IL-6 is a pre-requisite [12]; and downregulation of other pathogenic mechanisms, such as osteoclast activity [13].

### Addition of anti-IL-10 inhibits BiP function

Although BiP stimulates the production of IL-10 from peripheral blood mononuclear cells *in vitro *[1], we lacked histological evidence for the upregulation of IL-10 protein in the BiP-treated RASM/SCID mouse. This may have been due to the single time-point chosen for the experiment. To test the hypothesis that the therapeutic effect of BiP was at least partially mediated via IL-10, BiP or HSA were therefore simultaneously administered with either a neutralising anti-IL-10 antibody or an isotype antibody control.

**Figure 3:**
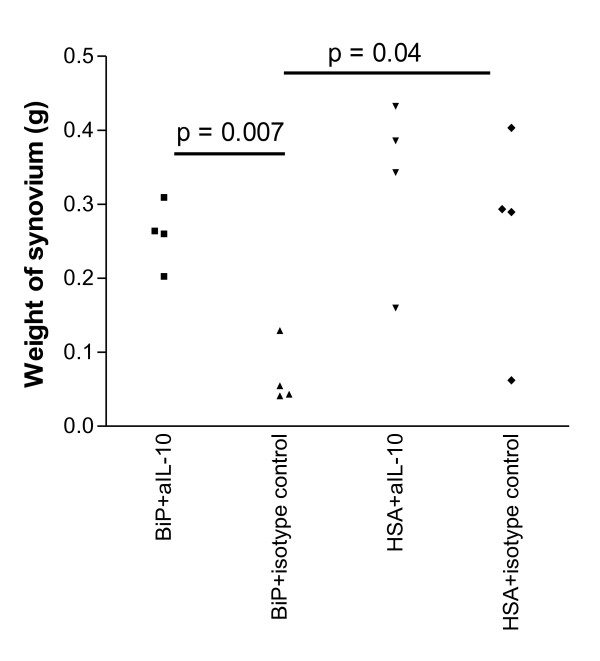
**Binding immunoglobulin protein attenuates inflammation in synovial membrane transplants via IL-10**. Equal sized pieces of rheumatoid arthritis synovial membrane (RASM) were transplanted into severe combined immunodeficient mice. After successful engraftment, binding immunoglobulin protein (BiP) (10 μg/animal) or human serum albumin (HSA) (10 μg/animal) were administered intravenously concomitantly with either a neutralising anti-IL-10 antibody (aIL-10) or isotype control antibody. The RASM graft was removed after 12 days and weighed as a surrogate measure of inflammation. The results of four animals in each group are shown.

The weight of the transplant was used as a surrogate measure of inflammation. Weight loss, indicative of the loss of inflammatory cells and reduced tissue oedema, signified a reduction of tissue inflammation. The explants from the mice given HSA, whether co-injected with the anti-IL-10 antibody or the isotype control, did not differ in weight (HSA + anti-IL-10, 0.33 ± 0.06 g; HSA + isotype, 0.27 ± 0.07 g; *P *= NS). In contrast, the explants from the mice given BiP + isotype control were significantly lighter in weight from those explants given anti-IL-10 (BiP *+ *isotype control, 0.07 ± 0.04 g vs. BiP + anti-IL-10, 0.26 ± 0.04 g; *n *= 4, *P *= 0.007) (Figure 3). Importantly, there was no difference in weight between the two HSA groups and the group given BiP + anti-IL-10 (Figure 3). These data provide indirect evidence of IL-10 involvement. Undoubtedly the production of IL-10 facilitates the attenuation of TNFα production [1], but data also indicate that the positive deactivation of monocytes [14] is important for the immunomodulatory effect of BiP, with downregulation of CD86 and HLA-DR and upregulation of soluble TNF receptor II and IL-1 receptor antagonist production [1]. These latter functions may be independent of IL-10 production [1,15]. It is certainly evident that, although BiP and IL-10 have a similar effect on monocytes, the kinetics are different [1] - therefore, at present, the extent to which IL-10 defines BiP activity is unclear.

Consideration should also be given to the extended activity of BiP treatment in the absence of protein. *In vivo *adoptive transfer experiments clearly indicate that the induction of regulatory T cells is important [2], and the identification of the regulatory cell subpopulation involved is currently under investigation. Likewise, the BiP-induced attenuation of IL-6 is a novel finding from this model and we are presently investigating this in relation to BiP.

## Conclusions

A single systemic administration of BiP in the RASM/SCID model post engraftment of the RASM suppressed all gross markers of pathology as recorded by Rooney and colleagues and by Koizumi and colleagues. In addition, CD86 and HLA-DR expression was reduced and IL-6 and TNFα production was attenuated. Evidence thus suggests that BiP resolves RASM inflammation and that this is at least in part a consequence of IL-10 production. The study confirms that BiP has long-term anti-inflammatory and immunomodulatory properties *in vivo*. There is therefore a reasonable expectation that BiP may prove to be an effective immunotherapy for RA acting by an entirely novel mechanism.

## Abbreviations

BiP: binding immunoglobulin protein; ELISA: enzyme-linked immunosorbent assay; HSA: human serum albumin; IL: interleukin; mAb: monoclonal antibody; RASM: rheumatoid arthritis synovial membrane; SCID: severe combined immunodeficient; RA: rheumatoid arthritis; TH: T-helper type; TNF: tumour necrosis factor.

## Competing interests

GSP and VMC are shareholders in the nonprofit-making company Immune Regulation Ltd, which holds the patent for the therapeutic use of BiP. KY, AO and HM have no conflicting interests.

## Authors' contributions

KY and AO carried out the animal work and the immunohistology. HM carried out the animal work and the immunohistology, participated in the design of the study and performed the statistical analysis. VMC and GSP performed the statistical analysis, conceived of the study, and participated in its design and coordination and drafted the manuscript. All authors read and approved the final manuscript.
